# Residual Symptoms After Carpal Tunnel Decompression and Treatment With Gabapentin: A Multicenter Study

**DOI:** 10.7759/cureus.17638

**Published:** 2021-09-01

**Authors:** Murat Aydin, Guldeniz Argun, Baver Acar, Murat Arikan, Güray Toğral, Selim Cinaroglu, Ahmet Mert, Mehmet Demi̇rtas

**Affiliations:** 1 Orthopaedics, Nigde Omer Halisdemir University, Nigde, TUR; 2 Anesthesiology and Algology, Ankara Oncology Training and Research Hospital, Ankara, TUR; 3 Orthopaedics, University of Health Sciences, Antalya Education and Research Hospital, Antalya, TUR; 4 Orthopaedics and Traumatology, Ankara Gazi University Medical School, Ankara, TUR; 5 Orthopedics, Dr Abdurrahman Yurtarslan Ankara Oncology Education and Research Hospital, Ankara, TUR; 6 Anatomy, Nigde Omer Halisdemir University Medical School, Nigde, TUR; 7 Orthopaedics and Traumatology, Nigde Omer Halisdemir University Medical School, Nigde, TUR; 8 Orthopedics and Traumatology, Hand and Upper Extremity Surgery, Memorial Hospital, Ankara, TUR

**Keywords:** gabapentin, carpal tunnel syndrome, neuralgia, decompression, surgical, pharmaceutical preparations

## Abstract

Objectives

To identify postoperative residual symptoms of carpal tunnel syndrome (CTS) and to investigate the effectiveness of gabapentin in the treatment of residual symptoms.

Materials and methods

Of a total of 412 patients who underwent surgery for CTS in four centers over a four-year period, 14 who had residual symptoms after CTS release and did not receive gabapentin (Group A) and 14 patients with postoperative residual symptoms and received gabapentin were included in this retrospective study. Postoperative residual symptoms were defined as persistent nocturnal numbness and tingling with or without occasional daytime pain. Tinel’s and Phalen’s tests were performed for the diagnosis of residual symptoms. Functional Severity Score (FSS), Symptom Severity Score (SSS), and Visual Analog Scale (VAS) were used to evaluate functional outcomes, severity of symptoms, and numbness and sleep quality, respectively at six and 12 weeks postoperatively.

Level of Evidence: III, therapeutic study

Results

There was no statistically significant difference in the mean postoperative FSS (p=0.845) and VAS-numbness scores (p=0.367) between the groups. However, there was a statistically significant difference in the mean postoperative SSS (p=0.025) and VAS-sleep quality scores (p<0.001) between the groups.

Conclusion

Gabapentin treatment can be a treatment of choice for residual symptoms after CTS surgery and clinical improvement can be achieved owing to its relieving effect, particularly in nocturnal symptoms of patients having neuropathic pain.

## Introduction

Carpal tunnel syndrome (CTS) is the most frequent entrapment neuropathy of the wrist [1,2]. Treatment of CTS includes the splinting and resting of the wrist, the use of non-steroidal anti-inflammatory drugs (NSAIDs), steroid injections, and surgical decompression [1-3]. The decompression procedure is performed using traditional open or endoscopic techniques with a high success rate. Although satisfactory results are reported in many patients following decompression surgery, there may be persisting symptoms [4]. In general, the complaints related to relapse are due to inadequate decompression of the transverse carpal ligament [5]. Several publications have shown that the risk of relapse following decompression varies from 1 to 31%; however, the rate of revision surgery is about 5% [5-7].

Gabapentin is an anti-epileptic agent [8]. It is a structural analog of gamma-aminobutyric acid (GABA), an important neurotransmitter in the central nervous system [9]. The United States Food and Drug Administration (FDA) first approved this agent in 1993 for the treatment of generalized and other partial epileptic seizures in patients over 12 years of age [10]. In addition to its antiepileptic efficacy, the effectiveness of gabapentin in other diseases was also shown in later studies. In the literature, publications associated with the use of gabapentin in indications other than epilepsy constitute about 40% of all gabapentin reports [8]. The main indication areas of gabapentin other than epilepsy are neuropathic pain syndrome, and psychiatric and movement disorders [8]. Chronic postoperative pain may occur following major surgical interventions (i.e., amputation, or hip replacement), as well as minor surgery (i.e., hernioplasty) [11]. Also, nerve defects developed during surgery may contribute to the chronicity. The NSAIDs, epidural or perineural local anesthetic, and opioid administration are frequently used methods for post-operative pain. However, these methods are not sufficient in many patients, particularly in those with chronic postoperative pain. In addition to these drugs, gabapentin and pregabalin have recently started to be used in the treatment of postoperative pain [12]. There are many studies investigating the effects of these agents on postoperative pain severity, morphine consumption, movement-related pain, chronic postoperative pain development, and intra- and postoperative parameters, as well as preemptive characteristics. In these studies, gabapentin doses varied between 300 to 1,200 mg, and pregabalin doses varied between 50 and 300 mg; a single dose was administered in most cases. In many studies, gabapentin or pregabalin significantly reduced the need for opioids [13-14]. During three studies related to long-term drug administration (two to 10 days) and long-term follow up (one to three months), the incidence of chronic neuropathic pain was found to be lower in cases of hysterectomy and mastectomy with gabapentin; however, another study showed no significant difference [15-17]. However, a recent study reported that gabapentin has no effect on postoperative pain after carpal tunnel syndrome [18]. According to these scientific studies mentioned above, gabapentin is effective in the treatment of chronic postoperative pain syndrome and neuropathic pain.

Following CTS surgery, some patients suffer from nocturnal numbness, tingling, pain, and occasional daytime pain. The severity of these complaints is usually milder than the preoperative period with negative Tinel’s and Phalen’s tests. In addition, based on our clinical observations, thenar atrophy and disrupted thumb kinematics during opposition are suggestive of severe median nerve compression and define postoperative residual symptoms of CTS.

To the best of our knowledge, there is no study identifying this patient group in the literature. In the present study, we aimed to identify postoperative residual symptoms of CTS and to investigate the effectiveness of gabapentin in the treatment of residual symptoms.

## Materials and methods

Study design and study sample

This multi-center, retrospective study was conducted at the Department of Orthopedics and Traumatology of four centers located in Turkey between January 2012 and December 2019. Written informed consent was obtained from each patient for all diagnostic and therapeutic procedures. The study protocol was approved by the Ankara Oncology Training and Research Hospital Ethics Committee (No. 2015-12/190). The study was conducted in accordance with the principles of the Declaration of Helsinki.

Medical records of a total of 412 patients with CTS who were operated on by five highly experienced surgeons from four centers were retrospectively reviewed. Exclusion criteria were as follows: the requirement of revision surgery due to relapse (n=7), pregnancy (n=2), lactation (n=2), known renal insufficiency (n=3), and hematological disease (n=1).

Seven patients (1.6%) were considered to be recurrent CTS as we reported relapse-related symptoms, such as persistent nocturnal and daytime pain and numbness, positive Tinel’s sign and Phalen’s maneuver on clinical examination. Electromyography (EMG) revealed an irreversible axonal defect in the median nerve in two patients and reduced transmission latency of motor and sensory fibers of the median nerve in five patients. Therefore they were not included in the study because of revision open surgical decompression due to CTS.

When evaluating differential diagnosis in the patient groups, peripheral polyneuropathy carpometacarpal arthritis of thumb, nerve tumors, cervical radiculopathy (C6), flexor carpi radialis tenosynovitis, pronator syndrome, Raynaud phenomenon, and pillar pain were not present [19-21].

The residual symptoms that the patients experienced and neuropathic drugs used following CTS surgery were retrieved from the hospital records. Among the screened patients with CTS who had decompression surgery (n=397 cases), 34 (8.56%) had persistent nocturnal numbness and tingling with occasional daytime pain, indicating postoperative residual symptoms of CTS, and experienced reduced clinical complaints during the postoperative period. The analysis of the medical records of these 34 patients showed that 19 patients did not receive gabapentin due to neuropathic symptoms after surgery (Group A), 14 of 19 patients without gabapentin usage were randomly selected in the study (Group A), while 15 patients received gabapentin for residual symptoms (Group B) (Table 1). Gabapentin had been discontinued in one patient due to drug intolerance (somnolence, weakness, dizziness, headache, nausea, vomiting) thus this one patient was not included in the study group (Group B). In case of drug intolerance due to side effects, the medication was continued by reducing each dose by 300 mg over three days. Gabapentin was discontinued in one patient due to drug intolerance, and this patient was excluded from the analysis. Fourteen patients who had nocturnal pain and numbness were retrospectively screened and they were assigned as Group B. Patient hospital records review were compared between these two groups retrospectively.

**Table 1:** Patients with neuropathic complaints (Group A, B) and patients who underwent revision surgery before starting gabapentin (Group A: patients who did not use gabapentin; Group B: patients who received gabapentin for residual symptoms) EMG: Electromyography

	Group A	Group B	Patients who underwent revision
N	14	14	7
Tinel’s sign	-	-	6 +
Phalen maneuver	1 patient + 13 patients -	2 patients + 12 patients -	7 +
Nocturnal Pain	10+	12+	7+
Daytime Pain	2 patients occasionally moderate	3 patients occasionally moderate or rare	4 +
Nocturnal numbness	14+ (often)	14+ (often, 1 patient occasionally)	7 +
Nocturnal tingling	(often, 1 patient occasionally)	14+ (often, 1 patient occasionally)	7+
Performance at hard work	12+ (None) 2+ ( Occasionally Feeling difficulty)	11+ (None) 3+ ( Occasionally Feeling difficulty)	7+,(Always Feeling difficulty)
Thenar atrophy (Preperative period)	8+	7+	
Opposition limitation of the thumb (Preoperative period)	2+ Carpal tunnel decompression plus modified Camitz opponensplasty	1+ Carpal tunnel decompression plus modified Camitz opponensplasty	
EMG	-	-	2; an irreversible axonal defect in the median nerve 5; reduced transmission latency

Among the patients receiving gabapentin for postoperative residual symptoms of CTS at six weeks, 14 patients were started gabapentin 600 mg/day b.i.d. as the initial dose. 14 patients (Group B) with residual symptoms of CTS were given gabapentin 600 mg/dose twice daily (the total daily dose was 1200 mg for each patient). Gabapentin was discontinued in one patient due to drug intolerance, and this patient was excluded from the analysis (somnolence, weakness, dizziness, headache, nausea, vomiting) thus this one patient was not included in the study group (Group B). The medical records of both patient groups were compared. Both groups were allowed to receive an NSAID (etodolac) 300 to 600 mg/day for approximately postoperative two or three weeks. The minimum duration of gabapentin use of the patients was 6 weeks. The maximum duration of gabapentin use of the patients was 49 months.

Assessment tools

The functional outcomes were evaluated using the Functional Severity Score (FSS). The severity of symptoms was assessed using the Symptom Severity Score (SSS). The Visual Analog Scale (VAS) was used to evaluate numbness and sleep quality. All assessments were performed at six and 12 weeks postoperatively. All four types of scoring were applied to cases in all four clinics. The visual analog scale is used to describe the subjective complaints of patients. Numbness of the hand and sleep quality were recorded and measured using a 10-cm visual analog scale (VAS), with the left end labeled “no numbness or good sleep quality” (0 cm) and the right labeled “very severe numbness or no sleep quality” (10 cm). We showed the patients the 10-cm ruler and asked them to mark the point along the ruler that represented their numbness or sleep quality severity. Therefore we used a scale of 0 to 10 cm, we rounded off in the usual method (e.g., 5.3 cm was rounded to 5 cm and 6.9 cm to 7 cm) [22-23].

Statistical analysis

The power analysis of the study was performed using the G*Power program. Between Group A and Group B for SSS scores at 12 weeks, an alpha (α) equal to 0.05 and a power (1-β) of 0.78 were assigned. Between Group A and Group B for VAS-sleep quality at 12 weeks, an α equal to 0.05 and a power (1-β) of 0.77 were assigned. Statistical analysis was performed using the SPSS for Windows version 25.0 software (IBM Corp., Armonk, NY, USA). Descriptive data were expressed in mean ± standard deviation (SD), median (min-max) or number and frequency, where applicable. The independent t-test was used to identify the differences between the groups. A p-value of <0.05 was considered statistically significant with a 95% confidence interval (CI).

## Results

Of the patients, nine were females and five were males in Group A and 10 were females and four were males in Group B. The mean age was 52.18±17.37 (range, 34-72) years in Group A and 53.24±19.36 (range, 32-74) years in Group B. There was no significant difference in the age and sex between the groups (p=0.21) (Table 2). One patient experienced headache and two patients had dizziness and dose adjustment was made due to these side effects, which all resolved following adjustment. At six weeks after surgery (prior to the initiation of gabapentin) and at 12 weeks postoperatively (six weeks after the gabapentin initiation), there was no significant difference in the mean FSS scores between the two groups, either (p=0.540 and p=0.845, respectively), indicating that gabapentin had no significant effect on the FSS scores during the postoperative period.

**Table 2:** Patients’ characteristics (Group A: patients who did not use gabapentin; Group B: patients who received gabapentin for residual symptoms) *Independent sample t-test; no statistically significant difference is shown.

	Group A	Group B	p-value
N	14	14	
Gender	9 F/5 M	10 F/4 M	
Age, years, mean±SD	52.18±17.37	53.24±19.36	.21*
Thenar atrophy	8	7	
Preoperative restriction of opposition	2	1	
Comorbidity	Diabetes mellitus (n=2)	Diabetes mellitus (n=2)	
	Hypothyroidism (n=0)	Hypothyroidism (n=1)	

In addition, there was no significant difference in the mean SSS scores was observed at the postoperative six weeks between the groups (p=0.761), six weeks after the initiation of gabapentin (Week 12), there was a significant difference in the mean SSS scores between the two groups (p=0.025). A significant improvement in the clinical scores with gabapentin was observed, as evidenced by the postoperative SSS scores in terms of nocturnal numbness and occasional complaints (moderate, tolerable).

There was no significant difference in the VAS-numbness scores at six and 12 weeks between the groups (p=0.786 and p=0.367, respectively). Gabapentin yielded no significant improvement in the clinical VAS scores in terms of nocturnal numbness and occasional complaints (moderate, tolerable), following CTS surgery.

Furthermore, at postoperative six weeks, there was no significant difference between the two groups for VAS-sleep quality scores (p=0.261). At postoperative 12 weeks, however, there was a significant difference in the VAS-sleep quality scores between the two groups (p<0.01). A significant improvement was achieved in clinical scores with gabapentin on the VAS-sleep quality scores in terms of nocturnal numbness and occasional complaints (moderate, tolerable), following CTS surgery (Table 3).

**Table 3:** Clinical scoring and statistical analysis (Group A: patients who did not use gabapentin, Group B: patients who received gabapentin for residual symptoms) FSS: Functional Severity Score; SSS: Symptom Severity Score; VAS: Visual Analog Scale.

	Group A	Group B	Independent samples t-test p values
FSS scores			
Week 6	1.53±0.38	1.45±0.42	.540
Week 12	1.54±0.47	1.59±0.60	.845
SSS scores			
Week 6	1.63±0.79	1.67±0.74	.761
Week 12	1.59±0.14	1.14±0.12	.025
VAS-numbness			
Week 6	20.5±10.57	18.13±8.67	.786
Week 12	21.46±10.51	16.30±6.46	.367
VAS-sleep quality			
Week 6	22.97±3.98	21.99±4.13	.261
Week 12	21.84±4.11	10.93±3.98	<0.01

## Discussion

In this study, we evaluated postoperative residual symptoms of CTS and investigated the effectiveness of gabapentin in the treatment of residual symptoms. In some of our patients, even after successful median nerve decompression surgery and the lack of any apparent etiological cause, nocturnal numbness, tingling and pain with occasional day time pain persisted. However, the results of the Tinel’s and Phalen’s tests were all negative.

Tung et al. classified secondary carpal tunnel surgery into three types as persistent, recurrent, and new symptoms following CTS surgery. Recurrent symptoms may continue or be new-onset following surgery [24]. The most frequent reason for persistent symptoms after surgery is incomplete surgical relaxation [25-26]. As surgical exploration of the distal transverse carpal ligament and proximal antebrachial fascia is difficult, the decompression of these structures by mini-open or endoscopic techniques may fail. Unquestionably, insufficient decompression is not the only cause and, additionally, fibrous proliferation and tenosynovitis may be the cause of incomplete release [27]. Therefore, although we performed conventional open surgery in all of our patients, we observed an intense fibrosis on the surgical field in all of our seven patients who underwent revision surgery and were excluded from the study. In recurrent CTS, there is a precise period of comfort following surgery; however, symptoms may manifest six months later. Epineurial fibrosis, interstitial scarring, and soft tissue adhesion are among the most frequent causes of recurrent CTS [28-30].

Previous studies including postoperative CTS patients have shown successful results following surgical decompression [31-34]. However, in spite of sufficient decompression, the rate of unsatisfactory results still remains high in CTS surgery. In a recent study, Louie et al. reported a mild, moderate, or severe persistent nocturnal pain in 7.4% and persistent numbness and nocturnal pain in 14.4% over a 10-year follow-up following CTS surgery (n=113) [35]. In our study, persistent symptoms were seen in 7.7%; however, the follow-up in the aforementioned study was made using the Levine-Katz Symptom Scoring scale, while we used FSS and SSS in our study. According to our study results, nocturnal pain and numbness were seen only in a small proportion of the patients (Of the 397 patients, 34 (8.56%) had residual symptoms).

In the literature, there is a very limited number of studies on the use of gabapentin for the medical treatment of CTS, and most have reported contradictory results. Taverner et al. (n=25) treated their patients with gabapentin at 1,800 mg daily dose for six months and observed a statistically significant decline in the symptoms and pain, although they found no significant changes in EMG [36]. In another prospective, randomized clinical study including 21 CTS patients, Duman et al. investigated the efficacy of gabapentin treatment and observed a significant improvement in the clinical scores in patients receiving gabapentin 600 to 900 mg/day for three months, compared to the control group; however, in that particular study, no data were available regarding the prognosis of patients following the discontinuation of the drug [37]. In an open-label study examining the efficacy and safety of gabapentin in CTS patients (n=41), Erdemoglu demonstrated that gabapentin at a daily mean dose of 1,800 mg was effective in the medical treatment of CTS at the end of six months; however, the lack of a control group is the main limitation of this study [38]. In another recent double-blind, randomized, placebo-controlled study with eight weeks of follow-up (n=140), gabapentin was not significantly different from placebo for the treatment of CTS in terms of the SSS scores [39]. Therefore, the efficacy of gabapentin in the medical treatment of CTS is still controversial due to both the limited number of studies and lack of comparative researches.

In our study, following 397 CTS operations, we created a group of 34 patients (8.56%) who had postoperative residual symptoms of CTS. However, revision CTS surgery was carried out in only 1.6% of the patients with positive EMG and physical examination findings. During 10-year follow-up period following CTS surgery, Louie et al. reported that mild, moderate, or severe nocturnal pain persisted ongoing in 7.4% and numbness and nocturnal pain persisted in 14.4% of the patients [35]. The higher incidence of these complaints in the study of Louie et al. compared to our study can be attributed to the longer follow-up period of the study and the use of a different scoring system [35].

Hesami et al. researched a study in 2018 on the treatment of carpal tunnel syndrome and gabapentin [40]. The cases were divided into four groups with different combinations of nocturnal splint, exercise and gabapentin. Carpal tunnel syndrome was treated medically only for one month. The combination of nocturnal splint, exercise and gabapentin improved VAS of pain and paresthesia compared to other dual combinations. However, there was no differences in the SSS and FSS score. In the present study, we observed four typical residual symptoms in CTS patients: nocturnal numbness, tingling, pain, and occasional daytime pain without daytime numbness or tingling, which are all milder than the preoperative period with negative Tinel’s and Phalen’s tests. In our study, gabapentin did not cause a significant change in the mean postoperative FSS and VAS-numbness scores in these patients, while there was a significant change in the mean SSS and VAS-sleep quality scores after CTS surgery. These findings indicate that, although gabapentin did not result in changes either to functionality or daytime numbness in patients with or without occasional daytime pain, nocturnal pain and numbness improved following CTS surgery. Of note, patients rated the VAS-numbness score at that time of measurement; however, gabapentin led to a clinical improvement in the mean SSS and VAS-sleep quality scores, as there was a significant improvement in the nocturnal symptoms. 

Nonetheless, there are some limitations to this study. Its retrospective design and the fact that operations were performed by multiple surgeons are the main limitations. The fact that all surgeons had received specialty training from the same university at different times, reduced our doubts about surgery and follow-up. Tugrul et al. reported that surgeons in the same country were similar in their approach to carpal tunnel syndrome [41]. Since all surgeons graduated from the same university and received hand surgery training with the same comprehensions at different times during the specialty training. The study design also precluded the creation of a placebo group as the control group and, therefore, only patients who underwent CTS surgery and were or were not prescribed gabapentin were compared. Another limitation of this study is the lack of the effect of gabapentin on EMG findings. In a previous study, however, gabapentin did not lead to any change in EMG recordings in patients with median nerve neuropathy [36]. Furthermore, there was no significant correlation between clinical and electrophysiological results of surgically treated CTS patients [42].

The main strength of this study is that, to the best of our knowledge, it is the first study to identify postoperative residual symptoms of CTS and to investigate the effectiveness of gabapentin in the treatment of residual symptoms.

## Conclusions

In conclusion, our study results suggest that gabapentin reduces postoperative nocturnal numbness and pain in patients who have occasional or persistent nocturnal numbness following CTS surgery. Patients who have residual postoperative carpal tunnel complaints can be given gabapentin 600 mg/day b.i.d. daily. We recommend using the treatment for at least 6 weeks. Based on these results, we can speculate that gabapentin is effective for neuropathic pain following CTS surgery. According to our clinical observations, improvement of these complaints occurs within a few days following the initiation of the drug. We believe that this study would pave the way for further studies which would provide an insight into the description and classification of neuropathic symptoms after CTS surgery.

## References

[1] Atroshi I, Gummesson C, Johnsson R, Ornstein E, Ranstam J, Rosén I (1999). Prevalence of carpal tunnel syndrome in a general population. JAMA.

[2] Gerritsen AA, de Krom MC, Struijs MA, Scholten RJ, de Vet HC, Bouter LM (2002). Conservative treatment options for carpal tunnel syndrome: a systematic review of randomised controlled trials. J Neurol.

[3] Cırpar M, Arı M, Türker M, Ekşioğlu MF, Cetik O (2011). [The efficacy and safety of limited incision technique in carpal tunnel release]. Eklem Hastalik Cerrahisi.

[4] Soltani AM, Allan BJ, Best MJ, Mir HS, Panthaki ZJ (2013). A systematic review of the literature on the outcomes of treatment for recurrent and persistent carpal tunnel syndrome. Plast Reconstr Surg.

[5] Langloh ND, Linscheid RL (1972). Recurrent and unrelieved carpal-tunnel syndrome. Clin Orthop Relat Res.

[6] Hulsizer DL, Staebler MP, Weiss AP, Akelman E (1998). The results of revision carpal tunnel release following previous open versus endoscopic surgery. J Hand Surg Am.

[7] Strickland JW, Idler RS, Lourie GM, Plancher KD (1996). The hypothenar fat pad flap for management of recalcitrant carpal tunnel syndrome. J Hand Surg Am.

[8] Magnus L (1999). Nonepileptic uses of gabapentin. Epilepsia.

[9] Palmer CM, Pope HG Jr Antiepileptic drugs. New Oxford Textbook of Psychiatry, Vol. 2.

[10] Susman N. (2000). Other anticonvulsants. Comprehensive Textbook of Psychiatry, Vol. 2, 7th ed..

[11] Akkaya T, Ozkan D (2009). Chronic post-surgical pain. Agri.

[12] Gilron I (2006). Review article: the role of anticonvulsant drugs in postoperative pain management: a bench-to-bedside perspective. Can J Anaesth.

[13] Mikkelsen S, Hilsted KL, Andersen PJ (2006). The effect of gabapentin on post-operative pain following tonsillectomy in adults. Acta Anaesthesiol Scand.

[14] Pandey CK, Sahay S, Gupta D (2004). Preemptive gabapentin decreases postoperative pain after lumbar discoidectomy. Can J Anaesth.

[15] Fassoulaki A, Stamatakis E, Petropoulos G, Siafaka I, Hassiakos D, Sarantopoulos C (2006). Gabapentin attenuates late but not acute pain after abdominal hysterectomy. Eur J Anaesthesiol.

[16] Fassoulaki A, Triga A, Melemeni A, Sarantopoulos C (2005). Multimodal analgesia with gabapentin and local anesthetics prevents acute and chronic pain after breast surgery for cancer. Anesth Analg.

[17] Fassoulaki A, Patris K, Sarantopoulos C, Hogan Q (2002). The analgesic effect of gabapentin and mexiletine after breast surgery for cancer. Anesth Analg.

[18] Sadatsune EJ, Leal Pda C, Cossetti RJ (2016). Effect of preoperative gabapentin on pain intensity and development of chronic pain after carpal tunnel syndrome surgical treatment in women: randomized, double-blind, placebo-controlled study. Sao Paulo Med J.

[19] LeBlanc KE, Cestia W (2011). Carpal tunnel syndrome. Am Fam Physician.

[20] Dengler J, Stephens JD, Bamberger HB, Moore AM (2020). Mimickers of carpal tunnel syndrome. JBJS Rev.

[21] van de Beek WJ, Schwartzman RJ, van Nes SI, Delhaas EM, van Hilten JJ (2002). Diagnostic criteria used in studies of reflex sympathetic dystrophy. Neurology.

[22] Kou W, Gareus I, Bell JD (2007). Quantification of DeQi sensation by visual analog scales in healthy humans after immunostimulating acupuncture treatment. Am J Chin Med.

[23] Zisapel N, Nir T (2003). Determination of the minimal clinically significant difference on a patient visual analog sleep quality scale. J Sleep Res.

[24] Tung TH, Mackinnon SE (2001). Secondary carpal tunnel surgery. Plast Reconstr Surg.

[25] Kulick MI, Gordillo G, Javidi T, Kilgore ES Jr, Newmayer WL III (1986). Long-term analysis of patients having surgical treatment for carpal tunnel syndrome. J Hand Surg Am.

[26] Gelberman RH, Pfeffer GB, Galbraith RT, Szabo RM, Rydevik B, Dimick M (1987). Results of treatment of severe carpal-tunnel syndrome without internal neurolysis of the median nerve. J Bone Joint Surg Am.

[27] Botte MJ, von Schroeder HP, Abrams RA, Gellman H (1996). Recurrent carpal tunnel syndrome. Hand Clin.

[28] Rose EH (1996). The use of the palmaris brevis flap in recurrent carpal tunnel syndrome. Hand Clin.

[29] Phalen GS (1966). The carpal-tunnel syndrome. Seventeen years' experience in diagnosis and treatment of six hundred fifty-four hands. J Bone Joint Surg Am.

[30] Assmus H, Dombert T, Staub F (2006). [Reoperations for CTS because of recurrence or for correction]. Handchir Mikrochir Plast Chir.

[31] Atroshi I, Hofer M, Larsson GU, Ornstein E, Johnsson R, Ranstam J (2009). Open compared with 2-portal endoscopic carpal tunnel release: a 5-year follow-up of a randomized controlled trial. J Hand Surg Am.

[32] Trumble TE, Diao E, Abrams RA, Gilbert-Anderson MM (2002). Single-portal endoscopic carpal tunnel release compared with open release: a prospective, randomized trial. J Bone Joint Surg Am.

[33] Ferdinand RD, MacLean JG (2002). Endoscopic versus open carpal tunnel release in bilateral carpal tunnel syndrome. A prospective, randomised, blinded assessment. J Bone Joint Surg Br.

[34] Jacobsen MB, Rahme H (1996). A prospective, randomized study with an independent observer comparing open carpal tunnel release with endoscopic carpal tunnel release. J Hand Surg Br.

[35] Louie DL, Earp BE, Collins JE (2013). Outcomes of open carpal tunnel release at a minimum of ten years. J Bone Joint Surg Am.

[36] Taverner D, Lisbona MP, Segalés N, Docampo E, Calvet J, Castro S, Benito P (2008). [Efficacy of gabapentin in the treatment of carpal tunnel syndrome]. Med Clin (Barc).

[37] Duman I, Aydemir K, Ozgul A, Kalyon TA (2008). Assessment of the efficacy of gabapentin in carpal tunnel syndrome. J Clin Rheumatol.

[38] Erdemoglu AK (2009). The efficacy and safety of gabapentin in carpal tunnel patients: open label trial. Neurol India.

[39] Hui AC, Wong SM, Leung HW, Man BL, Yu E, Wong LK (2011). Gabapentin for the treatment of carpal tunnel syndrome: a randomized controlled trial. Eur J Neurol.

[40] Hesami O, Haghighatzadeh M, Lima BS, Emadi N, Salehi S (2018). The effectiveness of gabapentin and exercises in the treatment of carpal tunnel syndrome: a randomized clinical trial. J Exerc Rehabil.

[41] Yıldırım T, Ünsal SŞ, Armangil M (2020). Comparison of Turkish and international trends in carpal tunnel surgery. Jt Dis Relat Surg.

[42] Bulut T, Sener U, Yağdi S, Kazimoğlu C, Sener M (2011). Relationship between clinical and electrophysiological results in surgically treated carpal tunnel syndrome. Eklem Hastalik Cerrahisi.

